# Improved Principal Component Analysis (IPCA): A Novel Method for Quantitative Calibration Transfer between Different Near-Infrared Spectrometers

**DOI:** 10.3390/molecules28010406

**Published:** 2023-01-03

**Authors:** Hui Zhang, Haining Tan, Boran Lin, Xiangchun Yang, Zhongyu Sun, Liang Zhong, Lele Gao, Lian Li, Qin Dong, Lei Nie, Hengchang Zang

**Affiliations:** 1 NMPA Key Laboratory for Technology Research and Evaluation of Drug Products, School of Pharmaceutical Sciences, Cheeloo College of Medicine, Shandong University, Wenhuaxi Road 44, Jinan 250012, China; 2 National Glycoengineering Research Center, Shandong University, Qingdao 266237, China; 3 NMPA Key Laboratory for Quality Research and Evaluation of Carbohydrate-Based Medicine, Shandong University, Qingdao 266237, China; 4 Shandong Provincial Technology Innovation Center of Carbohydrate, Shandong University, Qingdao 266237, China

**Keywords:** near-infrared spectroscopy, calibration transfer, piecewise direct standardization, improved principal component analysis

## Abstract

Given the labor-consuming nature of model establishment, model transfer has become a considerable topic in the study of near-infrared (NIR) spectroscopy. Recently, many new algorithms have been proposed for the model transfer of spectra collected by the same types of instruments under different situations. However, in a practical scenario, we need to deal with model transfer between different types of instruments. To expand model applicability, we must develop a method that could transfer spectra acquired from different types of NIR spectrometers with different wavenumbers or absorbance. Therefore, in our study, we propose a new methodology based on improved principal component analysis (IPCA) for calibration transfer between different types of spectrometers. We adopted three datasets for method evaluation, including public pharmaceutical tablets (dataset 1), corn data (dataset 2), and the spectra of eight batches of samples acquired from the plasma ethanol precipitation process collected by FT-NIR and MicroNIR spectrometers (dataset 3). In the calibration transfer for public datasets, IPCA displayed comparable results with the classical calibration transfer method using piecewise direct standardization (PDS), indicating its obvious ability to transfer spectra collected from the same types of instruments. However, in the calibration transfer for dataset 3, our proposed IPCA method achieved a successful bi-transfer between the spectra acquired from the benchtop and micro-instruments with/without wavelength region selection. Furthermore, our proposed method enabled improvements in prediction ability rather than the degradation of the models built with original micro spectra. Therefore, our proposed method has no limitations on the spectrum for model transfer between different types of NIR instruments, thus allowing a wide application range, which could provide a supporting technology for the practical application of NIR spectroscopy.

## 1. Introduction

Based on its ability to quantify the physical properties and chemical constituents of samples in one spectrum, near-infrared (NIR) spectroscopy has become a powerful tool for the industrial implementation of process analytical technology (PAT) [1,2,3,4]. Furthermore, establishing a calibration model is an indispensable, even decisive, part NIR spectroscopy’s application; therefore, constructing a robust model always needs to invest a lot of manpower and time. It is much more challenging for manufacturers to guarantee absolute consistency and stability when using two spectrometers. Unavoidable spectral deviation results in the degradation of the calibration model’s prediction performance. Furthermore, in a practical industry scenarios, different types of instruments are often used simultaneously, such as benchtop NIR spectrometers and portable instrument devices [5,6]. Admittedly, the miniaturization, portability, and low-cost characteristics of NIR devices are more conducive to NIR spectroscopy technology’s widespread application. Therefore, developing methods to transfer the existing model to new instruments with different signal-to-noise ratios, spectral resolutions, and wavelengths is a matter of considerable concern [7].

However, calibration transfer offers the possibility of dealing with models adapted to the new measurements by benefitting from the evolution of chemometrics algorithms, minimizing the effects of spectral deviation. Calibration transfer establishes a functional relationship between the source spectrometer and target instrument’s detection signals to ensure the consistency of their prediction results. Many model transfer methods based on different principles have been proposed in the literature. Slope/bias (S/B) correction can correct the prediction value when the relationship between spectral difference and prediction bias is linear [8]. In calculating piecewise direct standardization (PDS) [9], a linear relationship in a small window region can be established with the spectra obtained on two spectrometers. In addition, canonical correlation analysis (CCA) [10] could explore the correlation of two spectra sets rather than the covariance [11]. Eliminating the spectral differences through the conversion between two spectral spaces is the principle for spectral space transformation (SST) [12]. In addition, multi-level simultaneous component analysis (MSCA) [13] was developed to explore the underlying relationship of the multivariate data involving different factors [14]. The recently proposed parameter-free framework for calibration enhancement (PFCE) applies correlation constraints on the regression coefficients [15]. Alternatively, Tikhonov regularization [16,17] and domain-invariant partial least squares regression (di-PLS) [18] are both based on the original model coefficients. In summary, the commonly used calibration transfer methods can be roughly divided into three strategies: (a) the correction of prediction values (i.e., S/B), (b) the standardization of spectra (SST, PDS), and (c) the modification of model coefficients (Tikhonov regularization, di-PLS, PFCE) [14]. Moreover, spectral pretreatment algorithms, such as orthogonal signal correction (OSC) [19], multiplicative signal correction (MSC) [20,21] and finite impulse response (FIR) [7], have been studied regarding calibration transfer.

**Figure 1:**
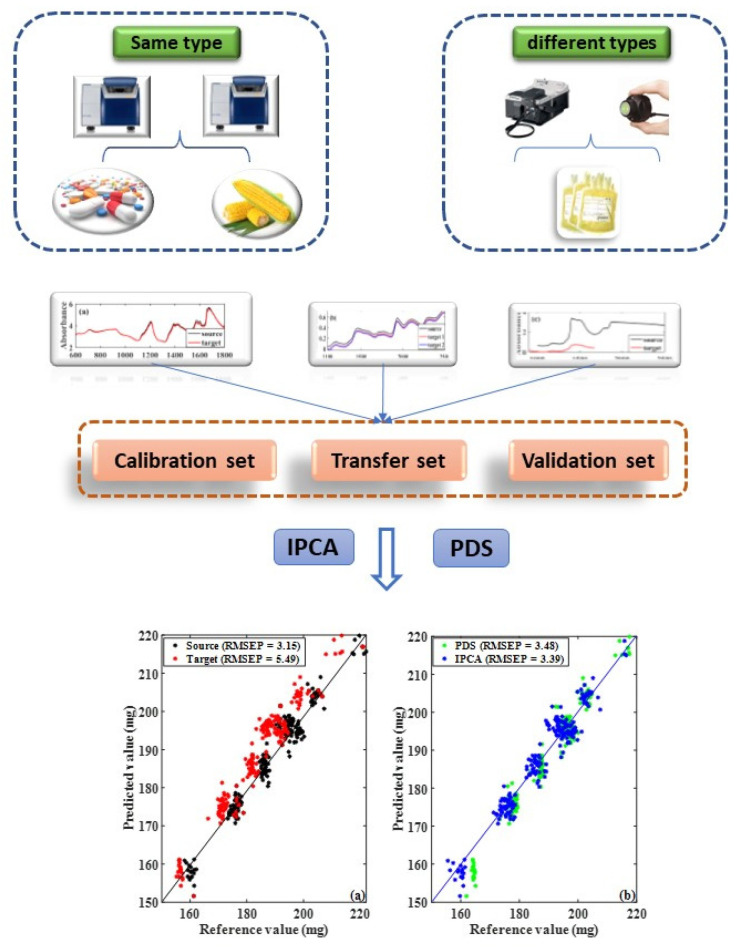
Schematic diagram of study.

Almost all of the methods mentioned above could achieve transfer between spectra with consistent data points, such as spectra acquired from two same-type spectrometers. However, few studies explored processing spectra with different data points and absorbance originating from different types of instruments [22,23,24,25,26], and PDS is still considered the most popular method. Therefore, a more practical approach to calibration transfer without requiring the same spectral data points is highly desirable. Here, we report a new algorithm inspired by principal component analysis (PCA) to map the responses obtained from one spectrometer to another. This method, named improved principal component analysis (IPCA), associates the source spectrometer’s spectrum data structure with the target spectrometer, converting a lower-resolution spectrum into a higher-resolution version (or vice versa), expanding the application of model transfer. Figure 1 shows our application of the proposed method to the NIR spectra of dataset 1 (public pharmaceutical tablets data), dataset 2 (public corn data), and dataset 3 (the spectra of eight batches of samples acquired from the plasma ethanol precipitation process collected by FT-NIR and MicroNIR spectrometers).

## 2. Results and Discussion

### 2.1. Calibration Transfer for Dataset 1

We utilized the spectra of the above-mentioned pharmaceutical tablet samples to establish a quantitative model for API content using the PLS regression method. Additionally, we adopted the leave-one-out cross-validation method to select the number of latent variables (nLV). First, we established a source model using the PLS regression method with the nLV set to 3, before constructing a transfer matrix with the samples in a transfer set by either PDS or IPCA. In the calibration transfer process, we corrected spectra in the target spectrometer’s validation set to fit for the source spectrometer with transfer matrix. Then, we predicted raw and transferred spectra in the validation set of the source and target spectrometer with the corresponding PLS model. Furthermore, we used the model’s RMSEP value to evaluate the transfer effect.

**Figure 2:**
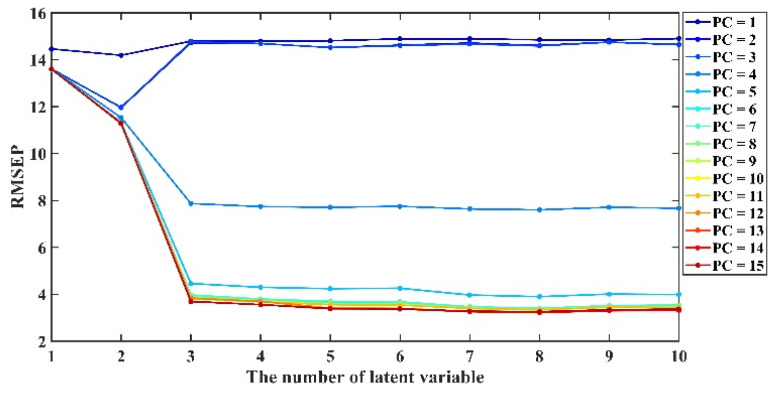
RMSEP results under different PCs and LVs with IPCA for transfer from target to source (different PCs in IPCA algorithm and different numbers of LVs in PLS regression).

The moving window width and nLV are vital parameters for PDS. Following a previous study [14], a moving window width set at 17 was optimal for PDS, while for IPCA, both the PCs during PCA and nLV are pivotal. Figure 2 shows a visualization of the impact of PC values on the model results, as well as the RMSEP values under different conditions in the calibration transfer from target to source, and there was a considerable difference in results under different LVs and PCs. When the number of PC was set to 1, 2, and 3, the RMSEP value did not change significantly across the entire range of LV values, which indicates that the spectral information’s structure cannot be comprehensively related at low PCs. When PC = 5, the RMSEP value decreases gradually with the increase in LV and stabilizes at about 8 after LV = 3. Additionally, when the PC is set from 6 to 15, the RMSEP value drops sharply at LV = 1–3 and stabilizes after LV = 4, which implies that the PCs play a considerable role during PCA and nLV; therefore, the appropriate choice of crucial parameters can achieve significant improvements in the predictive accuracy of IPCA.

**Figure 3:**
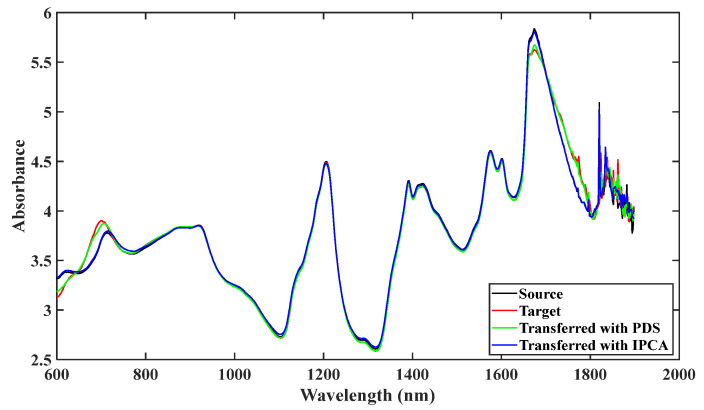
Raw and transferred spectrum of arbitrarily selected sample in dataset 1. Source, target, and spectrum transferred by PDS and IPCA represented by black, red, green, and blue curve, respectively.

**Figure 4:**
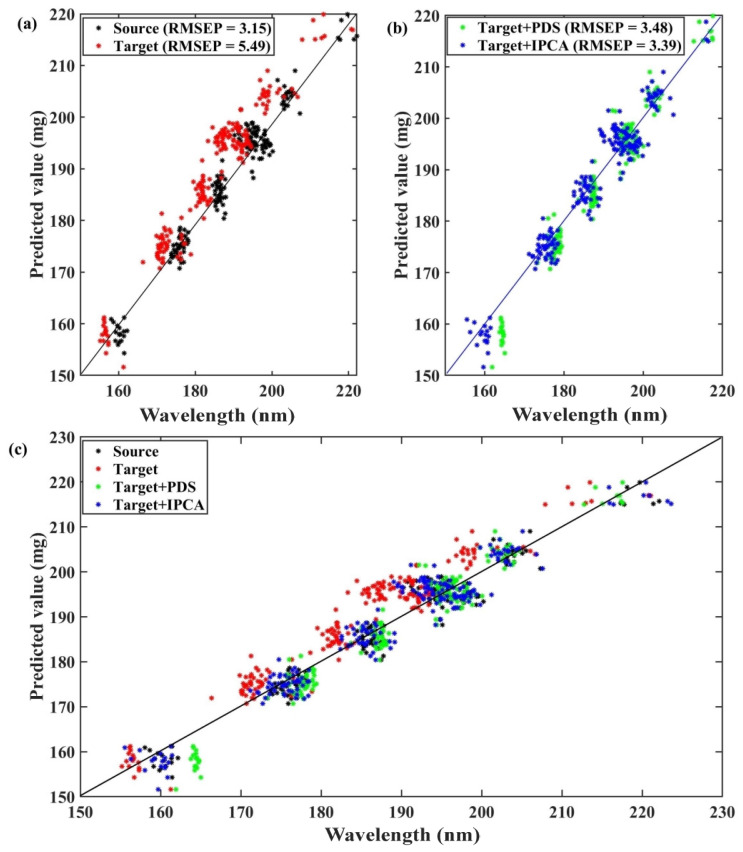
Relationship of reference and predicted API value in validation set obtained by (**a**) source and target spectra, (**b**) transferred spectra with PDS and IPCA, and (**c**) all of them, respectively.

To give more insights into the standardization effect, the spectra measured on the source, target and transferred spectra by PDS and IPCA for an arbitrarily selected sample are displayed in Figure 3. An obvious difference could be observed in the spectral regions of 600–700 nm, 1700–1800 nm, and 1800–1900 nm, and as mentioned above, the spectral ranges from 1800 to 1900 nm were cut off during model establishment owing to richness in noise. After being transferred with PDS, the target spectrum moves closer to the source spectrum with a difference in the range of 600–700 nm and 1700–1800 nm. The IPCA-transferred spectrum seemingly has better overlapping interval regions than the PDS-transferred spectrum. However, a slight difference could also be found in the wavelengths of 600–700 nm and 1700–1800 nm. Therefore, we speculate from Figure 4 that the transfer effect of IPCA is possibly better or comparable to that of PDS. We can infer that the IPCA method can achieve a better approximation of the source instrument spectrum by structurally associating the source instrument’s spectrum with the target instrument spectrum compared with the PDS method.

To give more insights into our proposed method’s evaluation effect, we utilized the source, target, and transferred validation spectra with the PLS model built with the source calibration spectra. The optimal nLV selected with the leave-one-out cross-validation in building the PLS model for the calibration set was 3, which generated the lowest RMSEP = 3.15 mg. This was in accordance with a common assumption that the source model acquired on the source spectra could lead to the best prediction of samples using their source spectra. The red points displayed in Figure 4a represent the relationship between reference and predicted values, showing a significant difference with the black points. That RMSEP = 5.49 mg (1.7 times larger than that from the source spectra) is further evidence that it is challenging to obtain model accuracy when the spectra collected by the target spectrometer are predicted by the model built with source spectra. Therefore, it was not feasible for the calibration maintenance to directly use the target spectra in the source model in a simple manner. Instrument difference is an important perturbation factor that cannot be ignored; therefore, appropriate calibration transfer is essential for improving accuracy. In our study, the 212 spectra in the validation set were transferred by PDS (window size was set at 17, nLV = 5) and IPCA, (nLV = 8, nPC = 10), and then predicted by the source model. In theory, the difference between the spectra of the target and source instruments can be eliminated as much as possible by standardizing the spectra with these two algorithms. Figure 4b shows that the RMSEP values decreased to 3.48 mg and 3.39 mg after calibration transfer with PDS and IPCA, respectively, indicating an improvement for the target spectra. The RMSEP value obtained with IPCA was slightly lower than PDS. In addition, the reference and predicted values of the IPCA method also had a higher overlap with the prediction results of the source spectrometer in Figure 4c. The clear deviation between the red and blue or green points was a visual representation of the target spectrometer’s improved prediction performance.

To further investigate the IPCA method’s performance, we listed the calibration transfer results with the interchanging the source and the target for dataset 1 in Table 1. The RMSEP of the target spectra predicted by the model was 3.41 mg, while the prediction of the source spectra was much worse (14.38 mg). The RMSEP value could be significantly reduced to 3.63 mg and 4.22 mg by calibration transfer with PDS and IPCA, respectively, where commonly used PDS produced a slightly better compared with the IPCA method. All the above results showed that both PDS and IPCA methods can be used for calibration transfer on the basis of meeting the following prerequisites: (1) Reasonable parameter settings are necessary as the too high or low selection of the nPC, nLVs or involved window sizes, caused a major performance hit. The parameter selection is also related to the user’s experience to some extent. (2) The number of samples in the transfer set also needs to be representative enough to ensure the transfer matrix’s reliability, and more than 30 representative samples are needed for this purpose.

### 2.2. Calibration Transfer for Dataset 2

**Figure 5:**
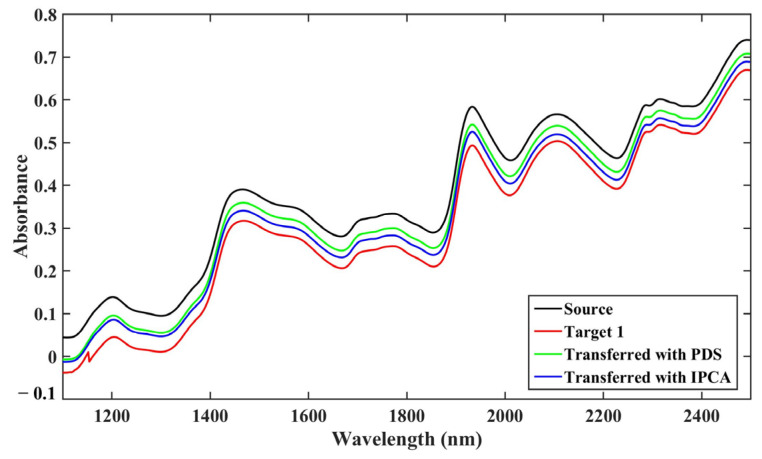
Raw and transferred spectra of arbitrarily selected sample in dataset 2 from source to target 1.

In a similar way, we utilized the spectra and oil content of corn samples in dataset 2 to establish the quantitative model. We adopted the source spectrometer’s 30 spectra in the calibration set to build a source calibration model; then, we adopted the source and target spectrometers’ 20 spectra (target 1 and target 2) in the validation set with/without transfer for model evaluation. The raw and transferred spectra of an arbitrarily selected sample from the source to target 1 are displayed in Figure 5. There are characteristic bands around 1400 nm and 1900 nm in the representative spectra of the source and target, which were likely assigned to the first overtones of O-H, N-H and C-H. In addition, the source spectrometer’s spectral absorbance is about 0.1 higher than that of the target instrument in the whole band. Because the source and target instruments’ representative spectra in Figure 5 originated from the same sample, we speculated that the difference in absorbance was mainly aroused by the instruments’ systematic errors. The spectrum absorbance was significantly improved by about 0.05 along with the wavelength after correction with PDS or IPCA. That means both methods could bring the spectrum closer to the source spectrometer spectrum and narrow the spectrum gap with the source instrument. Furthermore, the noise interference peak at 1150 nm was eliminated after correction. This indicated that the spectra quality could benefit from the calibration maintenance to some extent, reducing the spectral difference induced by the different instruments. It also means that the NIR spectra are vulnerable to the stability of instrument performance, which is the instrument manufacturer’s main responsibility.

The calibration transfer results obtained by both methods with interchanging the source and targets for dataset 2 are shown in Table 2. The target spectra of 20 validation samples were first corrected by the transformation matrix and then predicted with the source model, or vice versa. We found that when the established model was utilized for the prediction of the spectra in the validation set collected by the same spectrometer, the RMSEP values were at 0.1. However, an increase from 0.2 to 0.3 for RMSEP occurred when the established model was used to predict spectra obtained with different spectrometers. Therefore, we deduced that calibration transfer is crucial for model maintenance between different spectrometers of the same type. In addition, calibration transfer with PDS and IPCA could significantly reduce the RMSEP value by 0.1, whereby the IPCA method produced comparable results with commonly used PDS. The transfer results of dataset 1 and 2 in our study provided evidence that IPCA is a practical algorithm for model transfer between instruments of the same type, which could help reduce inter-instrument variance. However, the calibration transfer effect highly depends on the calibration, transfer, and validation of set samples. Public dataset 2 can be used for the preliminary verification of PDS and IPCA, although we need a larger number of samples for verification to provide more reliable results.

### 2.3. Calibration Transfer for Dataset 3

We analyzed dataset 3 to demonstrate our proposed method’s calibration transfer effect for spectra acquired from different types of spectrometers. Here, we take FT-NIR and MicroNIR as the source and target spectrometers, respectively, and their technical specifications are listed in Table 3. We observed that the two instruments have considerable differences in their principles of spectrophotometry, spectrum ranges, and resolutions, which also leads to distinctions between sample spectra.

**Figure 6:**
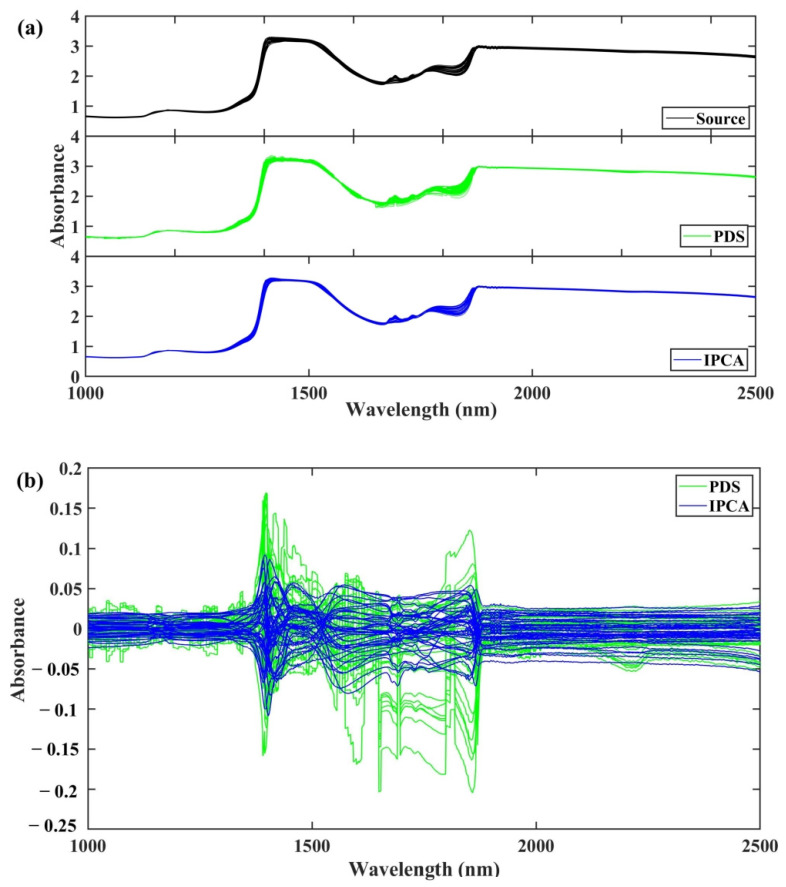
(**a**) Source (black) of transferred target spectra in dataset 3 with PDS (green) and IPCA (blue). (**b**) Different spectra obtained by subtracting original and transferred spectra in validation set with PDS (green) and IPCA (blue).

To obtain a further understanding of IPCA method, we investigated the calibration transfer between the two spectrometers in the whole spectral range in the following way: First, we converted the spectra collected by the target spectrometer with PDS and IPCA into the spectra of the source spectrometer in the range of 1000–2500 nm. In order to intuitively compare the transferred spectral differences between PDS and IPCA, we calculated the differences between the spectra by subtracting the transferred spectra from the source spectrometer’s original spectra. The source and transferred spectra, as well as their differences, are displayed in Figure 6. Figure 6a shows that after standardizing with IPCA from the target to the source instrument, the spectra are in excellent agreement with the corresponding spectra collected by the source instrument, which are relatively smooth in the whole spectral range. However, among the two methods, the transferred spectra with PDS have several small fluctuations in the 1000–1100 nm interval, as well as obvious large fluctuations in the 1400–1900 nm interval, especially at 1700–1800 nm, which may be caused by the large absorbance change intensity of the original spectra. This indicates that the quality of transferred spectra with PDS may be positively correlated with the original spectral fluctuation.

Although the transferred spectra are similar to that of the source instrument, there are still differences between them. The difference spectrum is an effective method to display the differences at each wavelength point. Figure 6b shows the spectra transferred by PDS and IPCA displayed different extent differences in the whole spectra range, with the largest difference in the 1400–1900 nm region. This suggests that the spectral variations brought by spectrometer differences could be systematic differences that vary from sample to sample [15]. The difference spectra also provide evidence that the IPCA may be more effective than PDS, as the difference spectra of IPCA were more stable in the whole spectral range, while the difference spectra of PDS varied along the wavelength. We deduced from the transferred and difference spectra that IPCA may achieve a better calibration transfer effect than PDS because the IPCA method’s dimensionality reduction procedure is able to eliminate some noise. All these results comprehensively demonstrated the effectiveness of the calibration transfer strategy with IPCA, especially in transferring spectra from low to high resolution.

**Figure 7:**
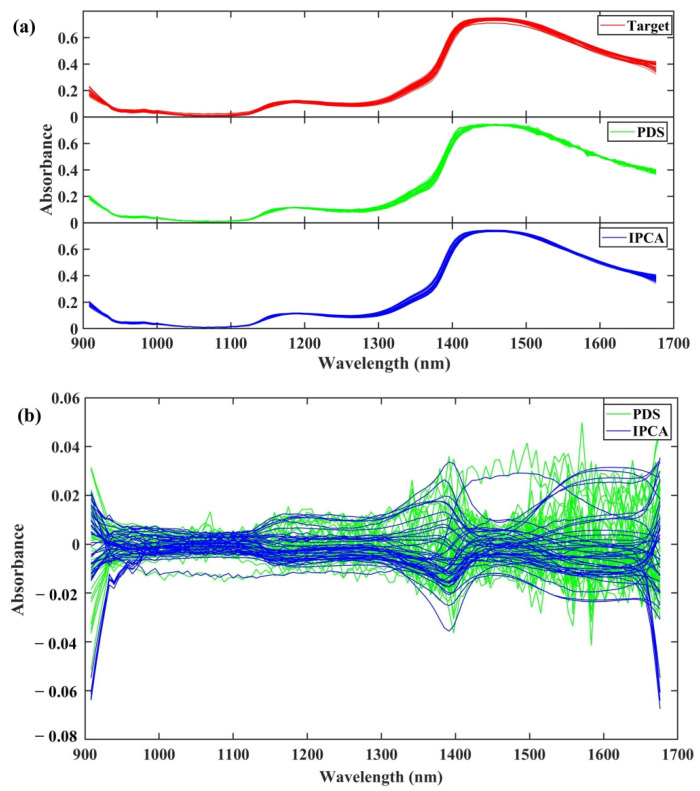
(**a**) Target (red) of transferred source spectra in dataset 3 with PDS (green) and IPCA (blue). (**b**) Different spectra obtained by subtracting original and transferred spectra in validation set with PDS (green) and IPCA (blue).

Similarly, the target spectra transferred by these two methods and the corresponding difference spectra are also displayed in Figure 7. We observed strong absorbance peaks around 1400 nm in both the spectra acquired from the source and the target spectrometers, which might be attributed to the first overtone absorption of O-H and N-H in the plasma ethanol precipitation process. Figure 7 shows that the spectra transferred with PDS were not as smooth as the spectra collected with the target spectrometer, especially the serrated bands that appeared between 1400 to 1700 nm, indicating a deteriorated spectral quality. However, the spectra transferred with IPCA were smooth and consistent with the original target spectra. The difference spectra obtained by IPCA were steady across the whole spectral range of 900–1700 nm, while the difference spectra obtained by PDS showed a relatively large value during 1400–1600 nm. Furthermore, there is a certain degree of deviation around the two ends (900 nm and 1700 nm) for the two methods, which may be due to the instability of both ends of the detector. Furthermore, compared with Figure 6b, the spectral difference threshold after the calibration transfer from the target to the source instrument is between −0.15 and 0.15, while the threshold for the opposite calibration transfer process is between 0.06 and 0.06. This indicates that in the calibration maintenance from the low- to the high-resolution spectra, the greater differences in the original spectra will cause larger deviations, or vice versa.

**Figure 8:**
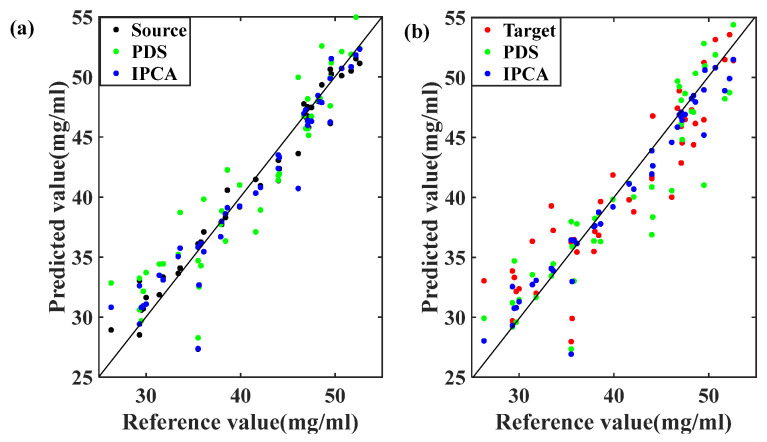
(**a**) Relationship of reference and predicted value in validation set obtained by (**a**) source—transferred target (PDS) or (IPCA), (**b**) target—transferred source (PDS) or (IPCA).

To further verify the IPCA method’s feasibility for calibration maintenance, we predicted the 42 spectra in the validation set after transferring with a PLS model built with the original spectra in a calibration set using the source and target. The bi-transfer results for dataset 3 are listed in Table 3 and displayed in Figure 8. Table 3 illustrates that the RMSEP values generated by PDS and IPCA were 2.78 mg/mL and 2.08 mg/mL, a 12% and 33% increase compared with the prediction error (3.15 mg/mL) acquired from the target spectrometer’s raw spectra, respectively. However, the RMSEP value generated by the transferred target (IPCA) decreased by 9% compared with the RMSEP value (1.89 mg/mL) predicted using the source spectrometer’s raw spectra, indicating a difference between the spectra before and after transfer. Furthermore, there is no significant improvement for spectra conversion in the whole spectral range with PDS, as the RMSEP value generated by the transferred source (PDS) was 3.13 mg/mL, which is almost equal with the RMSEP value (3.15 mg/mL) without transfer. Furthermore, the RMSEP value generated by the transferred source (IPCA) reduced to 1.90 mg/mL, indicating considerable improvements in the transfer from the source to the target spectrometer. We found that for different scenarios, the lowest RMSEP was yielded by spectra in the source spectrometer’s validation set, which was predicted with the PLS model built with the spectra in the calibration set. The target error could be improved by increasing the similarity between the source and target spectra, and IPCA generated better improvement. Furthermore, the scatter plot of reference and predicted value by IPCA was closer to the regression line than PDS in Figure 8. The IPCA calibration maintenance model showed a comparable or even better performance than the PDS models in accordance with the smallest RMSEP. Therefore, we speculated that IPCA was than PDS in improving the low-resolution spectrometers without common bands selection; therefore IPCA could be considered as an effective chemometrics means to optimize the prediction abilities of micro-instruments in practical use. Our findings demonstrated that the IPCA features better capture the signal that is related to the analyte and thereby eliminate noise to improve the model prediction effect [18].

To further verify our proposed method’s reliability, we performed a paired *t*-test under confidence intervals (CI) of 95% for the prediction results before and after the calibration transfer. In the paired *t*-test for two datasets, when P¼ is greater than 0.05, it means no significant difference and belonging to the same normally distributed population [27]. The corresponding P¼ results are also displayed in Table 4. In the comparison of the transferred target with the source, the P¼ of IPCA was far more than 0.05, which meant that there were no significant differences between the transferred target (IPCA) and original source prediction results. However, the P¼ of the PDS showed the opposite results. These results show that the IPCA method has superior performance in model transfer between miniaturized and benchtop spectrometers, especially in a transferring process from miniaturized (less data points) to benchtop spectrometers (more data points). However, the P¼ of both methods were greater than 0.05 in the comparison of the transferred source with the target, indicating no significant differences between the transferred source with original target prediction results. The results demonstrated that both methods could realize the transfer from spectra with more data points to spectra with fewer; however, the IPCA method generated fewer prediction errors than the PDS method.

Overall, the techniques employed in our investigation provided an adequate calibration transfer from primary to secondary instruments of the same and different types. Specific considerations will be presented for the proposed method, similar to PDS, the IPCA required approximately the same number of transfer samples from both spectrometers. The results obtained with IPCA were slightly better and comparable to those obtained with PDS in all cases (sharing the same spectral interval); however, they were better than PDS for dataset 3 in the whole spectral region. In addition, IPCA may be more convenient for subsequent use in routine analyses because it has no restrictions on data points. It is worth noting that PDS requires the adjustment of window size and IPCA needs the optimization of the number of PCs during PCA. In light of previous considerations, we deemed IPCA more appropriate for the cases under study. Our study for dataset 3 evaluates the feasibility of calibration models transferring between spectrometers with different types, especially between benchtop and handheld instruments, and the calibration transfer results of the handheld instrument are comparable to those of the benchtop instrument. The essence of calibration transfer is to correlate the spectrum of the source spectrometer with that of the target spectrometer, so that the model prediction ability after calibration transfer is greatly affected by the performance of the source and target spectrometers. As mentioned above, the number of spectral data points measured by the target and source spectrometers is 125 and 1557, respectively. Therefore, the calibration transfer process from target to source is an operation that could increase data points, which is equivalent to improving the spectral resolution. There is no doubt that this would considerably expand the application areas of handheld or miniaturized instruments or even realize the sharing of models between different instruments.

## 3. Materials and Methods

### 3.1. Theory and Algorithm

#### 3.1.1. IPCA

The IPCA method is based on PCA, which can associate the spectral data of the source spectrometer with the spectral data structure of the target spectrometer (or vice versa), suppressing noise or redundant features. Additionally, the IPCA method is capable of converting a lower-resolution spectrum to a higher resolution, solving the limitations caused by different wavelength points between spectra. Therefore, IPCA can realize bidirectional calibration transfer and forward (or backward)-IPCA transfer between the source spectrometer and the target instrument with no restriction on instrument type or whether the NIR spectra’s collected data points are consistent, thus considerably expanding the application scope of calibration transfer. The IPCA transfer process is described as follows:

We collected the corresponding **X_M_** and **X_S_** spectra with the source spectrometer and target instrument, respectively. We determined key parameters or reference values to be predicted with NIR spectroscopy using notational or industry standards. We adopted the same spectral preprocessing method, including but not limited to smoothing, derivative, standardization, etc., to preprocess the spectra collected by the two spectrometers, with no pretreatment considered as default.

Then, we selected a certain number of samples in the calibration set as transfer set samples to perform the calibration transfer. The number of transfer set samples measured by the target spectrometer was consistent with that of the source spectrometer. The number of transfer sets should be more than 10 but less than or equal to the calibration set; furthermore, our selection criteria was based on the minimum predicted root mean square error (RMSEP).

Then, we divided **X_M_** into calibration (**X_Mc_**), transfer (**X_Mt_**), and validation sets (**X_Mv_**) using an appropriate sample division method (K-S method).

Assume that the spectral matrices $X_{Mt}$ and $X_{St}$ are the corresponding spectra of the standardization samples in the transfer sets measured on both the source and target spectrometers, respectively.

Then, in the IPCA transfer, the singular value decomposition of **X_St_** can be displayed as below:

(1) $$X_{St}=\left[U_{St},U_{nt}\right]\left[\begin{array}{cc} \sum_{St} & 0\\ 0 & \sum_{nt} \end{array}\right]{\left[V_{St},V_{nt}\right]}^{T}=T_{St}P_{St}^{T}+E$$

where $T_{St}=U_{St}\sum_{St}$**;** $P_{St}=V_{St}$**;** $E=U_{nt}\sum_{nt}V_{nt}^{T}$**;** and $T_{St}$ and **P_St_** represent the principal component score and loading matrices of spectral data in the transfer set ($X_{St}$), respectively. Subscripts ‘**S**’, ‘**t**’, and ‘**n**’ represent the target spectrometer, transfer set, and noise, respectively. Superscript ‘**T**’ denotes the transpose operation.

In the IPCA method, the source spectra are not directly related to the target spectra; however, the $X_{\mathrm{Mt}}$ of the source spectrometer was straightforwardly associated with $T_{St}$ of **X_St_** of the target spectrometer under the condition that the number of principal components was set (see Equation (2)).

(2) $$T_{St}=X_{Mt}F_{Mt}$$

In Equation (2), $F_{Mt}$ refers to the transformation matrix, and the subscript “**M**” stands for the source spectrometer. Because $X_{Mt}$ is associated with $T_{St}$**,** there is no requirement that the spectral data points of $X_{Mt}$ and $X_{St}$ be consistent, and $T_{St}$ has the “best” explanation of $X_{St}$ based on PCA.

Equation (2) can be converted into $F_{Mt}=X_{Mt}^{+}\;T_{St}$ to calculate $F_{Mt}$, where “+” represents a generalized inverse operation and $X_{Mt}^{+}$ is the generalized inverse of $X_{Mt}$.

The spectra of the calibration set of the source spectrometer are transformed into spectra data suitable for the target spectrometer with a transformation matrix ($F_{Mt}$) based on Equation (3), which is obtained by substituting Equation (2) into Equation (1).

(3) $$X_{t-S}=X_{M}F_{Mt}P_{St}^{T}$$

where $X_{t-S}$ represents the spectrum matrix suitable for the target spectrometer after conversion. According to Equation (3), the spectra of the calibration set $X_{Mc}$ of the source spectrometer could be calibrated with Equation (4) into $X_{t-Sc},$ which is suitable for the target spectrometer.

(4) $$X_{t-Sc}=X_{Mc}F_{Mt}P_{St}^{T}$$

Finally, we adopted spectra in the validation set after transformation for model evaluation. The RMSEP value is the main evaluation index.

We carried out calibration transfer from target spectrometer to the source spectrometer in the reverse-direction transfer process, and then applied principal component decomposition on $X_{Mt}$. Then, we directly associated the spectra matrix $X_{\mathrm{St}}$ in the transfer set of the target spectrometer with $T_{\mathrm{Mt}}$ of **X_Mt_** under the condition that the number of principal components was set (see Equation (5)).

(5) $$T_{Mt}=X_{St}F_{St}$$

In Equation (5), $F_{St}$ refers to the transformation matrix. The subscript “**S**” stands for the target spectrometer.

Equation (5) could be converted into $F_{St}=X_{St}^{+}\;T_{Mt}$ to calculate $F_{St}$, where “+” represents a generalized inverse operation and $X_{St}^{+}$ is the generalized inverse of $X_{St}$.

The spectra of the target spectrometer’s calibration set are transformed into spectra data suitable for the source spectrometer with a transformation matrix based on Equation (6).

(6) $$X_{t-M}=X_{S}F_{St}P_{Mt}^{T}$$

where $X_{t-M}$ represents the spectral matrix suitable for the source spectrometer after conversion. According to Equation (6), the spectra of the calibration set $X_{Sc}$ of the target spectrometer can be calibrated with Equation (7) into $X_{t-Mc}$, which is suitable for the source spectrometer.

(7) $$X_{t-Mc}=X_{Sc}F_{St}P_{Mt}^{T}$$

If $X_{Sc}$ is a low-precision spectral matrix (such as the spectra scanned by a portable NIR spectrometer), then the conversion from low-precision to high-precision spectra **(**$X_{t-Mc}$, such as the spectra scanned by an analytical-level NIR spectrometer) can be successfully realized through $P_{Mt}^{T}$ in Equation (7).

#### 3.1.2. PDS

Variations in spectral data are often limited to small regions. Therefore, the source instrument’s spectral data points should be more related to the target instrument’s adjacent spectral points compared with the full spectrum. With spectral variation characteristics in mind, the PDS algorithm aims to reconstruct each spectral point on the source instrument with spectral data points in a small window.

In PDS, the response **r** of the standardization samples measured at wavelength/wavenumber j on the ‘source’ instrument is related to the wavelengths/wavenumbers located in a small window around j measured on the ‘target’ instrument:

(8) $$r_{j}=X_{j}b_{j}$$

where $X_{j}$ is the localized response matrix of the transfer samples and $b_{j}$ is the vector of transformation coefficients for the jth wavelength/wavenumber calculated by PLS.

Then, we formed a banded diagonal transformation matrix F as Equation (9),

(9) $$F=diag\left(b_{1}^{T},b_{2}^{T},\ldots ,b_{j}^{T},\ldots ,b_{m}^{T}\right)$$

where **m** is the number of spectral data points included in small windows. Then, the spectrum can be standardized with transformation matrix F.

### 3.2. NIR Datasets

**Figure 9:**
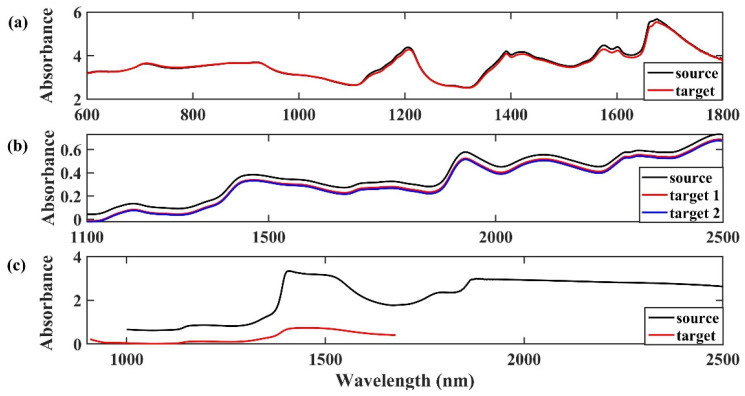
Typical source and target spectra of dataset 1 (**a**), dataset 2 (**b**), and dataset 3 (**c**).

We adopted three NIR spectral datasets to verify the IPCA method. Dataset 1 comprised 1310 spectra of 655 pharmaceutical tablets, which can be downloaded from https://www.eigenvector.com/data/tablets/index.html (accessed on 5 April 2005). We collected all spectra in the range of 600–1898 nm by two NIR spectrometers (Foss, Hillerød, Denmark) and assigned as the source and target spectra. We chose the active pharmaceutical ingredients (API) values for the method validation. Additionally, we selected total of 597 variables from 600 to 1792 nm for data analysis [28]. According to a previous study [14], we defined samples No. 19, 122, 126, and 127 in the calibration set and No. 11. 145, 267, 294, 295, 313, 341, 342, and 343 in the test set as outliers and eliminated them from our study. Then, we adopted the Kennard-Stone (KS) algorithm to divide the remaining 642 samples into a calibration and prediction set with 400 and 242 samples, respectively. Then, we randomly divided the 242 samples in prediction set into a transfer (30 samples) and validation set (212 samples). Figure 9a shows the typical spectra from the source and target spectrometers.

Dataset 2 comprised the NIR spectra of corn samples in the range of 1100–2498 nm acquired by three instruments (m5, mp5, and mp6), and can be downloaded from http://software.eigenvector.com/Data/Corn/index.html (accessed on 1 June 2005). We took m5 as the source instrument, while assigning mp5 and mp6 as the target instruments. In our study, we selected the oil content values for method evaluation. We divided the 80 samples into calibration (30 samples), transfer (30 samples), and validation (20 samples) sets using the above-mentioned methods. Figure 9b shows the typical spectra from the source and target spectrometers.

Dataset 3 comprised data acquired during the lab-simulated alcohol precipitation process of raw plasma (offered by Taibang Biologic Group). We placed each batch of 100 mL human plasma in a 250 mL container in a low-temperature reactor. We adjusted pH with acetic acid buffer to 5.95 ± 0.05 and added 95% alcohol solution at a constant speed. Then, we obtained 21 samples at every two minutes in one batch because the alcohol precipitation process lasted about 40 min. Then, we obtained 168 samples from eight batches. Moreover, we acquired the supernatant by centrifuging and filtrating all samples. We adopted the AU5800 automatic biochemistry analyzer (Beckman Coulter, Brea, CA, USA) for TP content determination. We used the FT-NIR (Thermo Fisher Scientific, Waltham, MA, USA) spectrometer as the source spectrometer. We collected the source spectra in the range of 10,000–4000 cm^−1^ (1000–2500 nm) of supernatant with the FT-NIR spectrometer (Thermo Fisher Scientific, Waltham, MA, USA) with a 4 mm pathlength cuvette in transmittance mode. Then, we assigned a miniaturized MicroNIR (Viavi, Scottsdale, AZ, USA) spectrometer with a spectral range of 950–1650 nm as the target spectrometer. We collected the spectra in transmittance mode with an integral time of 30,000 μs and a 1 mm optical path. We collected each sample three times at 26 ℃ and then averaged to reduce errors. We defined samples No. 12, 59, 76, 83, 86, and 100 as outliers and removed them from the experiment. We divided the 162 samples into 90, 30, and 42 as the calibration, transfer, and validation sets, respectively. Figure 9c shows the typical source and target spectra.

In our study, we collected the spectra of dataset 1 and 2 by the same type of near-infrared spectrometers. Figure 9a shows that changing the instrument resulted in obvious spectral variations in several spectral intervals, especially in the range of 1600–1800 nm. Figure 9b shows that the source spectrometer’s absorbance rate was higher than that of the target spectrometers. However, differences between instruments are unavoidable, which is why the calibration model is not suited for spectra collected by another instrument.

Figure 9c shows the spectra of dataset 3, which were acquired with different types of spectrometers, and the two spectrometers’ spectra display considerable differences in wavelength and absorbance. Based on the FT spectroscopic principle and high-sensitivity InGaAs detector, the source spectrometer’s spectral wavelength range from 1000 to 2500 nm, with absorbance between 0.5 and 3.5. Spectra collected by the target source with a linear variable filter (LVF) as an optical splitter and a 128-line-element InGaAs detector ranges from 908 to 1676 nm, with an absorbance level lower than 0.5. However, the overlapped spectral wavenumbers’ absorbance trends are similar.

We adopted Matlab 2019a (Mathworks, Natick, MA, USA) for data processing. We chose partial least squares (PLS) as the establishment method and selected the optimal PLS calibration model to have the minimal RMSEP value.

## 4. Conclusions

We proposed a novel calibration transfer algorithm for the transfer of NIR spectra acquired with different NIR spectrometers. Based on PCA, the IPCA could associate the source spectrometer’s spectral data with the spectral data structure of the target instrument (or vice versa) and suppress noise or redundant features, which makes it a flexible tool compatible with different model adaption situations. By adjusting the nPC in the calculation, the spectra taken on different spectrometers could be transferred between them. With public pharmaceutical tablets and corn datasets, we demonstrated that IPCA is capable of transferring spectra collected from different instruments with the same types of data points. Furthermore, the PLS model results provided evidence that IPCA is superior or comparable to the classical calibration transfer method PDS. We collected the spectra of eight batches of supernatant in dataset 3 by the benchtop FT-NIR and portable MicroNIR spectrometers, demonstrating that our obtained IPCA performance results are better than the PDS algorithm. Therefore, we believe IPCA can satisfactorily transfer NIR spectra collected on spectrometers of the same type or one miniaturization spectrometer to a benchtop spectrometer. Our model’s predictive capability after calibration transfer with the IPCA algorithm is better than the original source models. The IPCA algorithm can achieve model sharing between different instruments with the same and different resolutions. This even makes it possible for maintaining calibration models under different conditions, such as instrument, temperature, or other disturbances. To some extent, the PLSR model’s predictive capability was improved after calibration transfer. Our study is a useful resource for enterprises seeking use more cost-effective instruments for in-line or on-line process control. Moreover, IPCA could deal with spectral inconsistency and be applied to Raman, infrared, and other spectroscopy.

## Figures and Tables

**Table 1:** Calibration transfer results obtained by PDS and IPCA for dataset 1.

Calibration Spectra	Validation Spectra	Parameters	RMSEP (mg)
Source	Source	nLV = 3	3.15
	Target	nLV = 3	5.49
	Transferred target (PDS)	W ^a^ = 17, nLV = 5	3.48
	Transferred target (IPCA)	nLV = 8, nPC ^b^ = 10	3.39
Target	Target	nLV = 4	3.41
	Source	nLV = 3	14.38
	Transferred source (PDS)	W ^a^ = 17, nLV = 4	3.63
	Transferred source (IPCA)	nLV = 4, nPC ^b^ = 10	4.22

^a^ Window size selected in PDS. ^b^ Number of principal components selected in IPCA.

**Table 2:** Calibration transfer results obtained by PDS and IPCA for dataset 2.

Calibration Spectra	Validation Spectra	Parameters	RMSEP
Source	Source	nLV = 4	0.09
	Target 1	nLV = 4	0.24
	Target 2	nLV = 4	0.32
	Transferred target 1 (PDS)	W ^a^ = 17, nLV = 4	0.10
	Transferred target 1 (IPCA)	nLV = 5, nPC ^b^ = 4	0.17
	Transferred target 2 (PDS)	W ^a^ = 17, nLV = 4	0.13
	Transferred target 2 (IPCA)	nLV = 5, nPC ^b^ = 4	0.16
Target 1	Target 1	nLV = 4	0.09
	Source	nLV = 5	0.28
	Target 2	nLV = 5	0.14
	Transferred source (PDS)	W ^a^ = 17, nLV = 4	0.11
	Transferred source (IPCA)	nLV = 4, nPC ^b^ = 4	0.15
	Transferred target 2 (PDS)	W ^a^ = 17, nLV = 4	0.16
	Transferred target 2 (IPCA)	nLV = 4, nPC ^b^ = 4	0.16
Target 2	Target 2	nLV = 4	0.12
	Source	nLV = 4	0.27
	Target 1	nLV = 5	0.23
	Transferred source (PDS)	W ^a^ = 17, nLV = 1	0.18
	Transferred source (IPCA)	nLV = 1, nPC ^b^ = 4	0.17
	Transferred target 1 (PDS)	W ^a^ = 17, nLV = 4	0.15
	Transferred target 1 (IPCA)	nLV = 4, nPC ^b^ = 4	0.16

^a^ Window size selected in PDS. ^b^ Number of principal components selected in IPCA.

**Table 3:** Technical specifications of two NIR spectrometers.

Spectrometer	MicroNIR	FT-NIR
	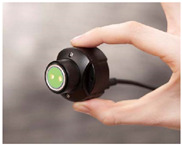	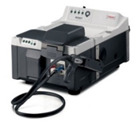
Spectral region	908–1676 nm	4000–10,000 cm^−1^ (1000–2500 nm)
Resolution	6.2 nm	4 cm^−1^
Wavelength filter	Linear variable filter	Interferometer
Light source	Two integrated vacuum tungsten lamps	Tungsten–halogen lamp
Sampling mode	Transmission	Transmission

**Table 4:** Results obtained by PDS and IPCA for dataset 3 in whole spectral range.

Calibration Spectra	Validation Spectra	Parameters	RMSEP (mg/mL)	Paired *t*-Test (CI = 95%)
Source (1000–2500 nm)	Source	nLV = 3	1.89	
	Transferred target (PDS)	W ^a^ = 17, nLV = 3	2.78	0.0303
	Transferred target (IPCA)	nLV = 3, nPC ^b^ = 4	2.08	0.9625
Target (908–1676 nm)	Target	nLV = 3	3.15	
	Transferred source (PDS)	W ^a^ = 17, nLV = 3	3.13	0.3241
	Transferred source (IPCA)	nLV = 3, nPC ^b^ = 5	1.90	0.3226

^a^ Window size selected in PDS. ^b^ Number of principal components selected in IPCA.

## Data Availability

The data used to support the findings of this study are available from the corresponding author upon request.
